# Supplementary material for: Parental experiences of the impacts of Covid-19 on the care of young children; qualitative interview findings from the Nairobi Early Childcare in Slums (NECS) project
Source: PLOS Glob Public Health. 2023 Aug 30;3(8):e0001127. doi: 10.1371/journal.pgph.0001127 (PMC10468034; doi:10.1371/journal.pgph.0001127)
Supplement: S1 Appendix — (DOCX) [file pgph.0001127.s001.docx]

# S1 Appendix: IDI Topic Guide and consent script

***Consent:***

| **No.** | **Script** | **Response and actions.** |
| --- | --- | --- |
| C1 | Good morning/afternoon/evening. My name is <NAME>, and I am a researcher from APHRC. We are conducting a research to find out how people who live in this area are managing during the COVID-19 pandemic. We are especially interested to learn about the care of young children.  Thank you for responding to the survey on [computer to automatically populate with date of CATI].  When we spoke with you then, we said we might call you back to ask some more questions.  Today, we want to ask if you would be prepared to talk to us more about your experiences over the last few months, and would like to, either today or in the next couple of days, to speak with you for about 30-45 minutes.  I would like to tell you more about the research so you can decide if you want to take part? Is that OK? | Y/N  If not interested, thank and end call. |
|  | This study is led by researchers from Kenya and the UK. We want to learn how you and your child/children are doing at the moment. We will ask about your household, your work and how your child/children is/are cared for, including whether this has been affected by COVID-19. The answers will be used to build an understanding of how COVID-19 is affecting people in this area.  This time, we would like to ask you some more open questions, and will give you more time to tell us in more detail about your experiences over the last few months.  The interview will take around 30-45minutes, and if you complete it you will receive 300KES of airtime to compensate you for your time. Participation is voluntary and you are free to stop the interview at any point, including part way through.  You and around 15 others were selected from those who completed the telephone survey in the last few weeks and who given their permission to be contacted for telephone research. We have chosen a variety of parents or guardians of young children who all live in Nairobi.  If you agree to participate your name, phone number and other personal details will not be shared with anyone. We will store your name so we can call you again in the future if you give us permission. Your answers, with all your personal details removed, and the answers of all the other participants may be shared with other researchers.  Results of the research, but not any of your personal details, will be shared in research reports or papers and with people who make decisions about child health and development policy in Kenya.  Do you have any questions? | Y/N |
|  | FAQs + answers:   - Why do you want to talk to me?   - We want to learn about you and your family, including how the coronavirus epidemics affecting your life. This is so that we can try to make sure that those who are trying to design programmes to help people like you understand the issues you face in your daily life. We are especially interested in people who are responsible for young children, as children can be especially affected by events around them. - Will I be paid for my answers?   - You will not be paid, but if you complete the survey, you will receive KES 300 of airtime to compensate you for your expenses, for example your battery use etc. - Why are you calling me again? I already answered your questions last time…   - We’re interested to learn a bit more about your experiences, and how the current situation, including COVID-19, is affecting you and your family. We’d like to ask some different questions, which focus more on your day to day experiences, and how you make decisions about care of your child/children at this time. - What happens if I change my mind about taking part?   - If you decide to take part, you are free to change your mind at any point, and for any reason. You can just say, and we will not contact you again, and we will delete any answers you have already given. - Who are you? And who are you working for?   - My name is [interviewer name]. I work for a group called APHRC. We are a research organisation based in Nairobi. For this project, we are working with researchers from APHRC (The African Population Health Research Centre) and the UK (the London School of Hygiene and Tropical Medicine & UCL). - Can you give me information about the virus?   - I’m afraid I cannot give you information myself, but I can help to direct you to reliable sources of up to date information. Would you like me to send a text message with some telephone numbers and websites? - Can you help me? I am struggling.   - I’m sorry to hear that. I’m afraid I cannot help directly. I can send you some information about ways to access help though by SMS? Would you like me to send a message with ways to contact organisations who are providing some support? - Whom can I call on further information about the survey?   - You can contact researchers at the African Population Health Research Center who can provide more information about the survey. I can give you their telephone number, and I will also send a SMS message after this call with their telephone number. - I have another question not listed here:   - You can contact researchers at the African Population Health Research Center who can provide more information about the survey. I can give you their telephone number, and I will also send a SMS message after this call with their telephone number. |  |
| C2 | [After answering questions:]  Would you like to take part in the research? | - Yes – now 🡪Questions - Yes - but, please call me back. 🡪Schedule call for later date/time - No 🡪Thank and end call. |

[All of above to be audio recorded, and available for audit as required].

| **Participant ID:** []  **Participant telephone number:** []  **Interviewer ID:** []  **Date/Time stamps (automated): START** [auto-populated]  **Date/Time stamps (automated): END** [auto-populated]  **Confirm consent given:**  [Y/N]  **Audio file:** [.mp3 or similar]  Respondent characteristics (from CATI interviews):   - Gender: M/F - User of paid childcare: Y/N - Relationship to child: - Name of youngest child: ______________   **Short description of the respondent (tone of voice, engagement and personality):**  [paragraph free text]  **Interviewer notes/reflections:**  [paragraph free text] |
| --- |

| **Section** | **Domains/Questions** | **Probes/Annotations** |
| --- | --- | --- |
| Introduction and tips: | - General purpose of the study - Aims of the interview - Expected duration – 30-45minutes - Who is involved in the process (other participants) - Why the participant’s cooperation is important - What will happen with the collected information and how the participant/target group will benefit - Confidentiality - Any questions? - Audio recorder – remind participants that you are recording the interview   **Instructions to facilitator:**  NOTE: The following is a guide. Try to ask all the questions below in the order given, but it is more important to maintain the flow of discussion. Suggested probes have been included.  **A note on probing:**  In-depth probing will allow you to clarify and illuminate responses given by a participant. Some examples include:   - Silence – allowing a participant to amplify what they have said - Mirroring – repeating back what the participant has just said   Repeating the respondent’s words as a question ie “milk is good for children?”   - Confronting the participant to clarify an earlier response “I’m a little confused, earlier you said that XYZ” - Using keyword probes – such as:  \| **Participant statement** \| **Moderator probe** \| \| --- \| --- \| \| It’s good \| What about it is good? \| \| I like the size \| What is it about the size? \| \| It is convenient \| In what way is it convenient? \| \| It works \| Can you tell me how it works? \|  - Using the third-person approach ie “you seem to have strong views on this. What do you think others might feel?” - Other probes include:   - Can you tell me more about that?   - What about that?   - What do you mean by that?   - What makes you feel that way?   - Can you think of an example of that?   - I’d like to know more about your thinking on that issue?   - I’m not sure I understand how you are using the word _____?   - What are some of the reasons for feeling as you do?   - You started to say something about…?   - You mentioned something about…?   The specific probes listed below are suggestions, but you do not need to be limited to them, and nor should you feel you need to ask all of them. Use your judgement, and try to allow the conversation to flow.  Remember – your job is not to teach, nor to judge. It is important that you do not look down on participants – you are trying to learn from them, not to inform or persuade them. We are looking for participants to take part and tell us what they know.  Suggested language for introduction:  *Good morning/afternoon. My name is <Ruth>, and I am calling today to learn about you and your family, especially the care of young children. I hope, over the course of the next 30-45minutes to learn from you a bit about your life, including how COVID-19 has impacted on you. Please feel at ease. There are no right or wrong answers.* | |
| Warm up [your household] | Can you tell us a bit about your child ?  (Habari ya leo, umekuwa aje tangu mwisho tulipokuzungumzia, kazi biashara?)Unaweza kutueleza machache kuhusu mtoto wako? (Familia yako je?) Ni nani anayeishi na wewe? Ilikuaje mpaka ukaja kuishi? | *Annotation: This question is mainly a ‘warm up’ to get the conversation going and to build rapport.* |
| ‘normal day’ | We are interested to know what your day to day activity looks like.  Thinking about a normal day, can you tell me how the day goes starting from when you wake up.  Tuna hamu ya kujua jinsi ambavyo shughuli zako za kila siku zinavyoendelea.  Ukiwaza juu ya siku ya kawaida, nieleze vile siku yako inaendelea kuanzia unapoamka *(Hasa ukizingatia mtoto/watoto wako, siku ya X inakuanga aje?)*  Probes:   - What do you do after that? - If mention childcare, what activities? Where?   Unafanyaje baada ya hiyo? | *Annotation: In the R2 quantitative survey we’re asking about who looks after children and where children spend their time, but are not exploring the parent/carer’s day to day activities, and how children and childcare fit in, including who looks after them (especially when they are busy) what sorts of activities children occupy themselves with.* |
| Childcare - who | Who are the different people who look after [Name youngest] over the course of a normal week?  Ni watu gani tofauti wanamtunza [Name youngest] katika wiki ya kawaida? (Nieleze kidogo kuhusu kila mtu anayeshughulika kumlinda X)  Probes:   - You? - Who else? - Sibling care? - Other family members? - Friends and neighbours? - Paid childcare? Nanny? | *Annotation: this open question is aiming to start an in-depth discussion about who cares for their youngest, where and why (ie how this fits in with other roles and demands on their time, or feelings about what is best for children). Probs should seek to explore all the different people who might be involved over a ‘normal’ week.*  *If conscious ‘choices or decisions between different options come up, try to probe how decisions are made, including who makes them and who influences them.* |
| Childcare journey over time | How has the care for [name] changed as they got older or over time?  Utunzaji wa [jina] umebadilika kwa njia gani anapo ongezeka umri au baada ya muda? (anavyoendele kukua unaona ni mambo yapi yanabadilika kwa kumtunza?)  Has that changed recently because of COVID-19? If so, how?Je, imebadilika hivi karibuni kwa sababu ya corona? Kwa njia gani?  [If uses paid childcare:  How did you choose this childcare provider?  ulichaguaje huu mtoa huduma za utunzaji wa watoto? (ni nini kilichokuvutia kwa mtunzaji?)  What is important when choosing a childcare provider? What do you need to look out for?]  Ni jambo gani lililo la muhimu unapo chagua mtoa huduma za utunzaji wa watoto? Ni kitu gani ambacho unahitaji kuzingatia? | *Annotation: This question seeks to explore the participants evolving childcare ‘jouney’ and how that is influenced by both the age of the child, and other factors like their work, or – more recently – the COVID-19 pandemic.*  *Try to listen and allow the participant to speak as much as possible, so that shifts or changes can be captured in their own words.*  *If they mention using paid childcare, try to explore how they found and chose their daycare provider; what was important to them in making that decision? How important was cost? Quality?* |
| Paid childcare | Please can you tell me about the daycares around where you live?  Tafadhali nieleze kuhusu vituo vya utunzaji wa watoto karibu na maeneo ambayo unayoishi? (ni mambo gani umeona/umeshuhudia,) | *Annotation: In this question, we want to explore respondents’ perceptions about paid childcare providers. Do not assume that either users and non-users of childcare have positive or negative views on the quality of daycare.*  *If they speak positively about it, try to to explore what they mean by ‘good’ quality.*  *Likewise, if they talk about ‘bad’ or substandard childcare, try to get them to unpack what that means to them “Can you tell me a bit more about what you mean by that?”*  *In addition, try to explore how these views on paid childcare impact on their own decision making about childcare.* |
| COVID-19 impacts | How has COVID-19 affected you and/or your family?  Please can you tell me a bit more?  Covid imekuathiri wewe kwa njia gani? Na familia yako je? Unaweza kunielezea zaidi? | *Annotation: This question aims to understand, including in ways that we may not have captured in the quantitative CATI questions, how COVID-19 has affected the respondent and their family. Again, it will be important to let them speak, and to let them guide the discussion. If needed, consider prompting them on any direct contact with COVID-19 for their family (themselves, or others becoming unwell) and indirect effects, for example on their work, day to day movement, or on community safety/violence, including domestic violence.* |
|  | How do you think COVID-19 has affected [name youngest]?  Covid imemuathiri [name youngest] kwa njia gani? | *Annotation: This question aims to build on the previous one, to ensure that the parent/carer’s views on impacts on the youngest child are captured, including any impacts on childcare arrangements. If this is already covered in the response above, no need to ask again.* |
| Closing | Thank you. Is there anything else you think is important that we have not talked about?  Asante. Kuna jambo lolote lingine ambalo ni muhimu na hatuja ligusia? jambo lingine ungependa kunieleza au kuuliza   - Summarise - Thank participant   Provide extra information and contacts to participants, including offer to send SMS with contact details for sources of information/advice.  After interview:  Data collector to:   1. Write a short description of the respondent (tone of voice, engagement and personality) 2. Write short reflective account on the interview/additional relevant context (e.g. “learnt loads of new tings”, ‘boring”, “surprising”, “confirmative”, “interrupted”, “mum shouted at child all the time” etc) | |
