# Supplementary figures and images for: Parental experiences of the impacts of Covid-19 on the care of young children; qualitative interview findings from the Nairobi Early Childcare in Slums (NECS) project
Source: PLOS Glob Public Health. 2023 Aug 30;3(8):e0001127. doi: 10.1371/journal.pgph.0001127 (PMC10468034; doi:10.1371/journal.pgph.0001127)

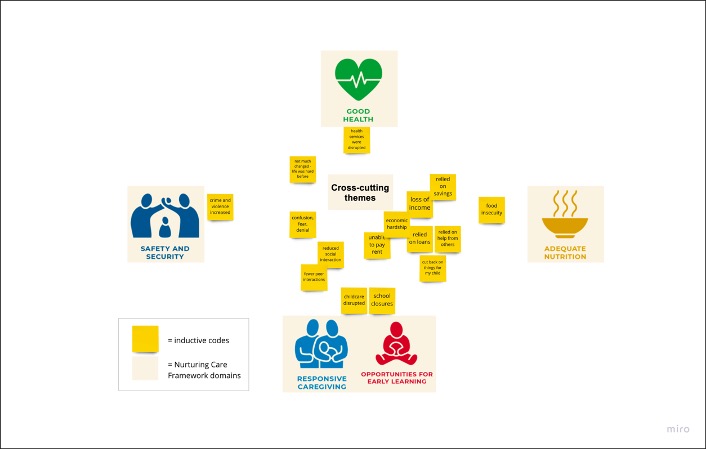

Supplement: S1 Fig — (JPG) [file pgph.0001127.s002.jpg]
