# Supplementary material for: Parental experiences of the impacts of Covid-19 on the care of young children; qualitative interview findings from the Nairobi Early Childcare in Slums (NECS) project
Source: PLOS Glob Public Health. 2023 Aug 30;3(8):e0001127. doi: 10.1371/journal.pgph.0001127 (PMC10468034; doi:10.1371/journal.pgph.0001127)
Supplement: S1 Table — (DOCX) [file pgph.0001127.s003.docx]

# S1 Table: Characteristics of IDI participants

## Table 1: Respondent characteristics

| **Characteristic** | **Frequency (n=21)** | |
| --- | --- | --- |
| Female | 13 |  |
| Male | 8 |  |
| Age of respondent   - 18-25 - 26-35 - 36-45 | 2  11  8 |  |
| Left formal education:   - During primary - Completed primary - During secondary - End of secondary - Post-secondary | 2  3  3  8  5 |  |
| Age of child/children   - 0-11m - 12-23m - 24-60m | 2  10  9 |  |
| Relationship to child   - mother - father - Grandparent | 11  8  2 |  |
| Use of paid childcare   - Yes - No | 11  10 |  |
