# Supplementary material for: Parental experiences of the impacts of Covid-19 on the care of young children; qualitative interview findings from the Nairobi Early Childcare in Slums (NECS) project
Source: PLOS Glob Public Health. 2023 Aug 30;3(8):e0001127. doi: 10.1371/journal.pgph.0001127 (PMC10468034; doi:10.1371/journal.pgph.0001127)
Supplement: Attachment — Submitted filename: NECS_Qual_PLOSGPH_Response to Reviewers_FINAL.docx [file pgph.0001127.s004.docx]

| **Section** | |
| --- | --- |
| **Comment** | **Response** |
| Journal Requirements: |  |
| Journal Requirements:  1. Please amend your detailed Financial Disclosure statement. This is published with the article. It must therefore be completed in full sentences and contain the exact wording you wish to be published.  a. State what role the funders took in the study. If the funders had no role in your study, please state: “The funders had no role in study design, data collection and analysis, decision to publish, or preparation of the manuscript.” b. If any authors received a salary from any of your funders, please state which authors and which funders. | Thank you; we have amended the Financial Disclosure Statement as follows:  This work was supported by the *British Academy (Grant number ECE190134) and Echidna Giving who supported RCH through a linked Clinical Research Fellowship. SB is supported by a NHIR clinical lecturership at Newcastle University.* BK, ZH, PK-W*, SO and RM received partial salary support from the British Academy grant, and RCH received partial salary support from Echidna giving. The funders had no role in study design, data collection and analysis, decision to publish, or preparation of the manuscript.* |
| 2. We have noticed that you have uploaded Supporting Information files, but you have not included a list of legends. Please add a full list of legends for your Supporting Information files after the references list. | Apologies for this omission; we have now added a full list of legends for supporting information after the references. |
| 3. In the online submission form, you indicated that "We are happy to make available to all qualified researchers on request the underlying data drawn on for this research. Given the challenges of genuine anonymisation of qualitative data, we have not posted the data to an open repository, but we are happy to facilitate sharing requests via the corresponding author.". All PLOS journals now require all data underlying the findings described in their manuscript to be freely available to other researchers, either 1. In a public repository, 2. Within the manuscript itself, or 3. Uploaded as supplementary information. This policy applies to all data except where public deposition would breach compliance with the protocol approved by your research ethics board. If your data cannot be made publicly available for ethical or legal reasons (e.g., public availability would compromise patient privacy), please explain your reasons by return email and your exemption request will be escalated to the editor for approval. Your exemption request will be handled independently and will not hold up the peer review process, but will need to be resolved should your manuscript be accepted for publication. One of the Editorial team will then be in touch if there are any issues. | Thank you; we can confirm that this approach to data management regarding the qualitative data was agreed with both the LSHTM and Amref Health Africa ethics boards in order to preserve anonymity of the research participants. |
| Editor Comments:   - The reviewers and I appreciate the potential for impact that this manuscript has, by conducting qualitative interviews to elucidate parental experiences during COVID-19 in low-income communities in Kenya. The manuscript is generally well-written and raises important questions. It also builds on and extends previous work in the communities in important ways. | Thank you; we agree that this is an important area which has, to date, received limited attention in the published literature. |
| - As noted by the reviewers, the methods need additional explanation. Given the prominence of the Nurturing Care Framework to the study design and analysis, it would be helpful to describe this framework in the introduction (perhaps a figure might also be useful), to help the readers understand how the framework informed the interview guide development and coding. Additional details on the interview guide development and revisions should be added, especially in terms of whether the guide was piloted, translated and back-translated, and how and why the guide was revised. I would also encourage the authors to include their final interview guide and codebook as appendices/supplemental materials. | Thank you (and the reviewers for this comment; we fully agree and have added (1) additional description of the framework (at lines 94-98), and (2) an additional figure (Figure 1) to illustrate it and to help orientate the reader.  In addition, as described below we have expanded on the methods section to explain how the interview guide, and how it was used in interviews, evolved. We have explained how it was drafted, translated, back-translated and then discussed amongst the team before being used and then iterated on.  I can confirm that the appended interview guide was the final version used. In addition, we have also added a supplementary figure, illustrating how the codes that were inductively derived and how they were related to the Nurturing Care Framework Domains, referred to at line 242-244 (Supplementary Figure 1). |
| - The comment that a phenomenological approach was used is a little confusing. I did not see reference to bracketing in the analysis, nor was it clear how deep, lived experience might be understood by a 15 minute phone interview--perhaps the authors also included data from the parent study to gain a deeper understanding of participants' experiences? If so, this should be made clearer. Further, the research is strongly driven by an external framework (the Nurturing Child Framework), which also seems somewhat counter to a phenomenological approach. I think the pragmatic approach makes more sense, unless there is additional information that I missed. | Thank you; we agree that it’s a bit confusing as written; we have clarified the methods to explain our pragmatic epistemological position. |
| - I also agree with Reviewer 2 that some of the assertions made in the discussion (child-centered data, gendered differences) are not very clear in the results. In addition, the links between the Nurturing Care Framework and the themes could also use additional explanation. | Thank you; we’ve worked to clarify all of these points as outlined in more detail below. |
| - I would also encourage the authors to consider Reviewer 1's questions related to the potential benefits/utility of the research to the participants, and to be a little clearer about the community engagement piece. If the community engagement methods and outcomes are outside the scope of the manuscript, then perhaps these are already described in a separate paper or could be added to the supplementary materials. I also wondered whether research participants received any financial incentive or phone airtime for their participation. | Thank you; we completely agree that these issues are of critical importance. We have attached as a new attachment to the paper an **author reflexivity statement**, which discusses these issues in more detail, and have also added additional detail about the community engagement work, including how we’re working to describe both the processes of these and the outcomes and next steps towards – hopefully – contributing towards positive change regarding the deficiencies and adversities we described, in forthcoming papers.  In addition, we have clarified that participants were compensated for their battery use etc through a small transfer of airtime after completion of call – see lines 228-231.  *Participants were provided with a modest (equivalent to USD3) talk-time credit after completion of the interview, as a contribution towards their expenses, for example battery charging for their phone.* |
| Reviewer #1: |  |
| This article is an offshoot of a wider project, begun before covid, about childcare in Nairobi townships. The authors used their database and contacts to carry out additional research about the impact of covid on households with young children. Whilst the research methods are impeccable, and the exposition is very clearly stated, this article raises the ethical questions put forward by, for example, Seye Abimbola about foreign gaze and structural inequities. The research reveals the shocking circumstances of families barely surviving in their day to day life. But who is such research knowledge for, and where is it being reproduced? What is the benefit to the participants of having participated? The article mentions "community involvement" and "community meetings" but gives not details. Instead, the authors state that "Consideration of these insights can help to inform mitigation efforts and future epidemic control policy discussions." If this is the main purpose of the research, how is this to be done? Who with? What role will those interviewed play in future mitigation efforts, other than having given information about their circumstances? I would be happier with this article if the ethics of a mainly white Western Research Institution (LSHTM) working with extremely deprived women and the cliches about policy discussions, were more stringently explored. | Thank you for your positive feedback regarding the clarity of our methods and articulation of the research questions.  In addition, we fully agree with the importance of the issues raised by Dr Abimbola and others. We are – as a collaboration between researchers in the global North and South - are very mindful of the concerns you raise, including questions about how such studies can/should inform changes to these circumstances. As we’ve described in more detail elsewhere (<https://bmjpaedsopen.bmj.com/content/4/1/e000822> & <https://royalsocietypublishing.org/doi/full/10.1098/rstb.2020.0430>), the findings reported here are part of a wider effort to build a broad understanding of the opportunities to improve early childhood in slums through rigorously describing the current realities.  In terms of who this work is for, we hope that it can inform both local policy making and intervention design (for example through our ongoing efforts to convene key stakeholders; a description of which we have expanded on at lines 210-218), and also regional/global efforts, including the newly established World Bank Transforming Childcare Fund (https://www.worldbank.org/en/topic/education/brief/childcare-incentive-fund). We will also expand on these points considerably in a forthcoming ‘synthesis’ paper which will draw together the qualitative and quantitative findings of the whole programme of research with a focus on policy implications and uptake.  In addition, given our focus in this paper on the pandemic, and policy responses to it, we hope that awareness of these experiences can also inform wider discussions on pandemic preparedness and response. In terms of the makeup of the research teams and our institutions, we’ve discussed these in some detail in the **author reflexivity statement,** but we agree they are vital. Although LSHTM, due to structural biases/tied aid restrictions of the funder (the UK Government via the British Academy) is the lead applicant on the grants which primarily supported this work, the research team is mostly female, mostly non-white and 50% Kenyan. |
| Reviewer #2: | Thank you for your thorough and detailed review, and for the numerous suggestions, the vast majority of which we agree with and have tried to incorporate into the revised manuscript. |
| Introduction • Please make sure you use the same word consistently e.g. covid, Covid-19, covid-19, covid19, etc | Thank you; we have harmonized all of these to Covid-19 throughout. |
| • This needs more detail on the specific public health and social measures that were enforced as they differed in each country. Was the focus on social distancing, re-direction of public healthcare services to COVID-19, closure of business, restriction of movements, etc. The kind of measures that were enforced will be associated with the impact and can therefore frame these impacts. What were the impacts of covid measures on social determinants of health in general in Nairobi/Kenya? | Thank you; we agree that this context is important. We have added an additional description of the evolving pandemic control measures that were enacted in Kenya in the period before data collection at line 132-143:  In addition, we have directed the reader to a more detailed chronology as described by Herman-Rolloff et al.  *At the time of the interviews, C~~c~~ovid-19 control measures were ongoing, albeit much less stringent than early in the pandemic. These consisted of night curfews (from 8pm and then later from 10pm), mask mandates in public areas, limits on public gatherings and advice to work from home (11). In the preceding months much more stringent controls were in place, including widespread closures of workplaces and schools, ‘stay at home’ orders, movement restrictions and re-organisation of public health services towards Covid-19 treatment; a detailed chronology of these evolving controls is described by Herman-Rolloff et al. (12).* |
| • Specify the age for early childhood | Thank you; we agree that it’s useful to be more specific about this (sometimes differently defined) term, so have added the following at line 98-100:  *While early childhood is described differently by different organisations, for the purposes of this research we have focused on the period until a child’s fifth birthday.* |
| • What were the direct impacts of Covid-19 on children in Nairobi/Kenya/Africa? Please give a few lines on findings from research on covid impact on children in Nairobi/Kenya/Africa. | Thank you; we have added the following, at line 107-111:  *The direct effects of Covid-19 on children in Kenya, and the wider region, are poorly documented due to limitations in testing, reporting and healthcare systems (6). That said, overall, the direct effects – despite the prevalence of immunodeficiency due to malnutrition and HIV – appears to be limited, with a recent UN Inter-agency Group for Child Mortality Estimation concluding that the direct effects of covid on child mortality ‘remain very mild’(7)* |
| • The word “slum” can be considered derogatory and stigmatising. Please replace it with a non-stigmatising word. | While we appreciate that the term can be seen in this way, at the same time, others, as for example Ezeh et al, (in the Lancet Series on The Health of People who live in Slums <https://www.thelancet.com/series/slum-health>) have argued that it’s important to distinguish slums from more neutral – but different – terms like informal settlements in recognition of the specific features of slums including neighbourhood effects. In addition, during recent public engagement work with these communities that has been completed by some of the authorship in Nairobi community members expressed a preference for the term slum; indeed they felt that the term informal risked being more stigmatising.  In view of this, we would prefer to retain the term. |
| Methods |  |
| • The parent study needs to be introduced here and the link between both studies. | Thank you for this suggestion; we’ve discussed this amongst the team, but felt that introducing the wider study in the Introduction (as at line 124, and directing readers to the protocol paper and commentary) was, on balance, clearer. |
| • Specify which ethnicities | Thank you for this suggestion; we have added information on the most prevalent ethnic groups at line 154-155, as below:  *All three slums are well established and have existed for decades. The ethnicity across all is mixed (including significant populations of Kikuyu, Luo, Luhya, and Kalenjin) as is the mix of new arrivals (including rural-urban migrants and international migrants) and long-standing residents. Each slum is loosely divided into villages, which tend to be dominated by one ethnic group, but boundaries are frequently blurred.* |
| • what was the sample size and how did you decide on this sample size e.g. Malterud's information power | As noted at line 249, we conducted 21 interviews.  We also describe at line 197 how we, through approximately weekly team meetings discussed how approach saturation determined when to end the data collection. |
| • line 149: reference for the literature review. Is this an existing literature review or was the review conducted by the authors of this article prior to the study? | Thank you for this comment; we have clarified that this was based on a rapid review of the emerging literature: (line 168-169)  *This topic guide was informed by a rapid review of the emerging literature on Covid-19 and nurturing care review, including the review conducted by Shumba and colleagues(5) which considered how the domains of the Nurturing Care Framework (Health, Nutrition, Responsive caregiving, Early learning, and Security and Safety) would be likely to be affected by Covid-19 and/or pandemic control measures.* |
| • Introduce the framework in the introduction | Thank you for this suggestion; we agree that this will help to guide the reader; we have added the following to the introduction, and also included an additional figure (Figure 1) to introduce the Nurturing Care Framework in the introduction at line 95-98:  *Early childhood is a critical window of opportunity; adversity in this period is a central social determinant of health and wellbeing, affecting later life learning, earning and happiness (4), The 2018 joint WHO/UNICEF/World Bank “Nurturing Care” Framework (illustrated in Figure 1), describes five – intersecting – domains or components which can support healthy early childhood development: good health; adequate nutrition; responsive caregiving; opportunities for early learning; and safety and security (5). While early childhood is described differently by different organisations, for the purposes of this research we have focused on the period until a child’s fifth birthday.~~.~~* |
| • Line 158: is this the NECS study? Needs more detail. | Thank you; we’ve amended this line as follows to clarify that this list was from the NECS Covid impacts sub-study, and to make the reference to the sub-study protocol more clear:  *First, a list of respondents who had completed up to 5 rounds of the NECS Covid impacts tracker sub-study, (a bi-monthly structured telephone surveys tracking the impact of Covid-19 on the care of children in slums (12)) was randomly ordered.* |
| • What was included in the information script? | Thank you for this suggestion; we have included reference to this in the text (at line 222) and have also included the full information script and FAQs within Appendix 2.  *At the start of interviews, an information script including the rationale for the study, the voluntariness of participation, and information on data handling/sharing (See Appendix 2) was read out and participants were asked to confirm that they agreed (1) to take part, (2) for the conversation to be recorded, translated and transcribed, and (3) for these data and results to be shared and used with researchers and others both in and outside of Kenya.* |
| • Did all participants consent? Could participants decline to participate? How many declined? | Thank you; we have clarified that interest in participation was determined though the recruitment call, and included information on the number who were reached but declined to participate, and their reasons: (line 193-199):  *Timing of telephone interviews was pre-arranged through a recruitment call, during which respondents indicated whether they wanted to take part in the research; amongst those reached, none declined to participate after the study was explained to them. For the interview itself, participants were asked to find a quiet place to take the call. Calls started with RM introducing herself and reading participant information and consent scripts. Where necessary this information was re-phrased to explain it clearly to participants to ensure understanding and any emerging questions were answered by RM.* |
| • Please include a section on researcher reflexivity i.e. critical reflection about the position you are taking as a researcher, and the influence of your specific background, education, race, gender, socio-economic status, etc in relation to the target group in your research, and how you have taken this stance into account in your research. | Thank you for this suggestion; we agree it’s important, and have expended on the description of our backgrounds, and how it informs our approach to this work, at lines 250-260:  *RM, SO and PW are mixed methods early childhood development researchers living and working in Kenya. RCH, ZH, SB and BK are UK-based child health and development researchers. RCH has worked as a health adviser at several international donor organisations. SB is a practising community child health physician. All authors, being based in albeit inter-disciplinary, health research organisations bring biomedical experience/perspectives to this research, although they all work on social determinants of health.*  In addition, we have added the following to explain how we, as a team, reflect on our stances in this work:  *Our frequent meetings to discuss fieldwork and themes allowed us to reflect on the data as a team which, given our varied backgrounds and experiences, allowed us to reflect on how our backgrounds informed our interpretations. These included reflective discussions with RM about how her positionality, especially conducting interviews by telephone, how this may impact respondents and how this could be mitigated, for example through considering the timing/scheduling of calls, investing appropriate amounts of time in introductions and building rapport.* |
| • Also include CREDIT author statement. | Thank you; we have amended the Author Contributions Section as follows:  RCH, BK, ZH & PK-W conceptualised the NECS study and RCH, PW & SB the linked Covid-19 tracker study. RCH led the design of the methods, data handling of the transcriptsand analysis for this paper and wrote the first draft, with supervision from ZH. RM conducted the interviews, coordinated transcription and checking of transcripts and reviewed the draft manuscript, before discussing it with RCH and then all authors. RCH, RM, PK-W, SO and ZH regularly discussed and reviewed analytical coding as the data was collected. All authors edited this draft and provided important intellectual content. All authors approved the final manuscript. |
| Results |  |
| • Line 234: reported direct effects | Thank you; we have added ‘reported’ here. |
| • Please give some detail on the reported direct effects as an introduction. Which direct effects did the study ask about and what was the response? | Thank you for this suggestion; we have added some addition detail on the direct effects of covid (in terms of child illness) in the introduction – at line 104-109, and have also clarified that the lack of direct effects being reported came from an open question about how the pandemic had affected families, rather than directly asking about covid infections: (line 292-296)  *However, all of the effects were indirect; although we did not directly ask about Covid-19 infections, when asked how the pandemic had affected their lives, none of the interviewees described knowingly suffering from Covid-19 infection themselves, or their children becoming unwell with the disease.* |
| • Line 272: Was this not already the situation before COVID-19? Were men now more responsible for earning money? This doesn't really show that the effects were gendered. Life before Covid-19 was most likely already divided along gender lines. How did it change during Covid-19? Did these divisions become more entrenched? And how exactly? | Thank you; we agree that this could be more clearly described; we have re-written this section as follows:  *These indirect effects of the pandemic were influenced by gender too. Male respondents frequently reported being especially responsible for earning money for the household, and females, including girls not attending closed schools, were more commonly responsible for childcare. Respondents described how the upheaval caused by the pandemic exposed some of these pre-existing expectations, and in some cases disrupted them, for example the crisis necessitating both parents to earn money for the household.* |
| • Separate the “early learning” and give it its own section. | Thank you for this suggestion; we had in fact started with this as a separate section in an earlier draft of the paper. However, we subsequently decided that, due to the very blurred boundaries between responsive caregiving and early learning in the early years, it made more sense to combine them. |
| • Did the parents/carers report how this increase in domestic violence affected the children? For example, did children show an increase in behavioural issues or ran away from home or started bed wetting, etc? How did it affect the children? | Thank you for raising this question; We agree that it is important; we have added the following to clarify that that the way that these changes affected children was not described in our interviews. (Line 442-444)  *In interviews, perhaps due to the sensitive nature of this topic and the taboos which sometimes affect it, respondents did not report if or how this increase in domestic violence affected children.* |
| • What are your additional insights? Point them out specifically. Right now, this section reads as if you only found what others have also found. What did your study add? | Thank you for this suggestion; we agree that we could do this more clearly. We have added several points to the discussion in the revised manuscript, attempting to pull out more clearly how our study provided rich descriptions of the – often more generally described – impacts that have been modelled/estimated elsewhere, including the specific setting of a slum, and the particular ways in which young children have been impacted. |
| • Another limitation is that children themselves were not interviewed. How did they experience the restrictions? | Thank you; we agree, and have added the following at line 558/559:  *We were also unable to remotely interview children themselves to ask them directly about their experiences.* |
| • What do you mean by fragile and weak systems? Are you referring to the healthcare system, the informal economy, social services, the schooling system, etc? Specify what you are referring to. | Thank you; we agree, and have amended at line 589-591to clarify this:  *This includes through disrupting fragile and weak health, education, childcare and (largely informal) employment systems, and through this placing considerable economic and social distress on vulnerable families and communities.* |
