# Supplementary material for: Parental experiences of the impacts of Covid-19 on the care of young children; qualitative interview findings from the Nairobi Early Childcare in Slums (NECS) project
Source: PLOS Glob Public Health. 2023 Aug 30;3(8):e0001127. doi: 10.1371/journal.pgph.0001127 (PMC10468034; doi:10.1371/journal.pgph.0001127)
Supplement: Attachment — Submitted filename: Response to reviewer comments 2.docx [file pgph.0001127.s005.docx]

Response to reviewer comments 2

Dear Dr Brault,

Thank you, and the reviewers for these further comments/suggestions. Please find a point-by-point response, and attached clean and tracked changes versions of the manuscript.

Best wishes,

Rob and co-author

| **Comment** | **Response** |
| --- | --- |
| *Editor’s comments:* |  |
| First, instead of using participant ID numbers to differentiate statements made by different participants, I think it would be more helpful to provide basic demographic information (for example, male caregiver, 30 years old, etc.). | Thank you; we agree that this will help the reader, so have added these to each quote in the first instance where that respondent is quoted, reverting to the participant IDs subsequently. |
| Second, Reviewer 3 raises a question about the age of the children and behaviors discussed by parents. This is not a major revision, but might be helpful to clarify if possible. | We agree that this is an interesting question; we have addressed it below, and made a short addition to the discussion to reflect this too (at line 555-556). |
| *Reviewer 3* |  |
| Although with relatively little data (21 participants who were interviewed for between 14 and 39 minutes) this is a well conducted and reported study. Please note- I didn't review the earlier version. | Thank you. |
| I was surprised that the authors were focused on young children under 5, as many of the quotes seemed to be about life in general, or about school aged children, for example "Playing is good but she was not reading at all. So, the things she had learnt, the teacher had to teach her again so she can catch up… she lagged a little." There are not many children who do a lot of reading before their 5th birthday, so strange to choose this quote, rather than anything about delayed language learning, development of confidence, experience of the world etc.  Similarly: "Corona has affected them because they were playing as a group outside… So now you know he plays alone in the house so he is bored." I wouldn't have thought many little children (aged 0-2) would be playing in a group outside, pre-covid, or that they would be particularly bored inside.  There was nothing that seemed related to having babies or young toddlers at home (for example, if there was no work outside the house, did this give opportunities for longer breast-feeding?), but given this was within a larger study on paid childcare, perhaps the focus was actually on pre-school children i.e.: aged 3-5? Rather than <5? Or is it possible that parents were recruited based on their younger child, but ended up discussing more about the ways the lockdowns were impacting their older school-aged children (perhaps because the impacts were bigger on those older children). | Thank you for raising this question; we agree that it’s an interesting one, and also that many responses may well reflect/relate to broader impacts on families, including those of older children. We have added this point to the discussion at line 555-556.  In addition, we have clarified that this quote about lagging at school was from a caregiver to an older child (who is now at school), so may have related to school-readiness. Furthermore, as suggested by the editor, we have added further detail to the quotes beyond the participant IDs, to aid the reader.  Related to young children playing outside; this is perhaps an interesting example of the context being relevant. In our experience of working in these settings it is quite common for young children (including the four-year old referred to in this quote) to play outside, often with limited adult supervision. |
| This is the only concern I had. Otherwise this looks good for publication. | Thank you! |
